# Laser- Written Freestanding Electrodes Based on Laser-Reduced Graphene Oxide/Carboxymethylcellulose for Non-Invasive On-Food Paraquat Detection

**DOI:** 10.1021/acsomega.6c03514

**Published:** 2026-07-17

**Authors:** Vinicius N. B. Souza, Vitor H.N. Martins, Maria H. Verdan, Cecilia C. C. Silva, Silvia M. Martelli, Victor H.R. Souza

**Affiliations:** † Faculty of Engineering, Universidade Federal da Grande Dourados (UFGD), Dourados, MS 79804-970, Brazil; ‡ Faculty of Exact Science and Technology, 186079 Universidade Federal da Grande Dourados (UFGD), Dourados, MS 79804-970, Brazil; § MackGraphe, Mackenzie Institute for Research in Graphene and Nanotechnologies, Mackenzie Presbyterian Institute, São Paulo, SP 01302-907, Brazil; ∥ School of Engineering, Mackenzie Presbyterian University, São Paulo, SP 01302-907, Brazil

## Abstract

The advancement of sustainable precision agriculture necessitates high-performance, accessible sensing platforms capable of in-field operation. Herein, a scalable, solvent-free Laser Direct Writing (LDW) strategy is reported for fabricating freestanding, seamlessly integrated electrochemical devices based on Laser-reduced Graphene Oxide/Sodium Carboxymethylcellulose (LrGO/CMC) biocomposites. Uniquely, the CMC acts not merely as a matrix but also chemically stabilizes GO sheets via hydrogen bonding, preventing restacking and enabling photothermal processing to selectively convert the film surface into an expanded, highly conductive LrGO architecture. Meanwhile, the unmodified bulk preserves the composite’s mechanical integrity. This continuous architecture, in which the active electrode is chemically derived from the substrate itself, effectively eliminates interfacial delamination, thereby enabling flexibility under dynamic stress. Analytically, the device leverages the high electroactive surface area of the rough LrGO network. Using optimized Differential Pulse Voltammetry (DPV) coupled with a rapid electrochemical conditioning step, the sensor demonstrates sensitivity to the Paraquat herbicide (limit of detection of 5.7 ± 1.2 μmol L^–1^ and a limit of quantification of 18.7 ± 4.1 μmol L^–1^) and is suitable for field monitoring, while showing selectivity against complex matrix interferents. As a ″lab-on-a-fruit″ proof-of-concept, the flexible device was successfully applied to the rapid (<1 min), noninvasive detection of pesticide residues directly on orange peels using a hydrogel interface. This work establishes LrGO/CMC composites as a versatile and resource-efficient platform for next-generation wearable electronics.

## Graphical abstract

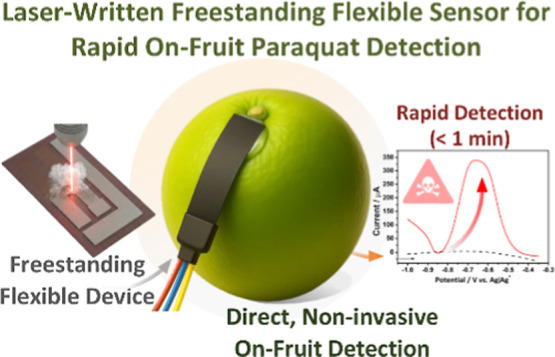

## 1 Introduction

The rapid intensification of global agriculture, driven by demanding food supply chains, has led to a paradigm shift in agrochemical consumption, reaching 3.5 million tons in 2021.^1–5^ To mitigate the environmental and health risks associated with indiscriminate pesticide use, the transition to precision agriculture necessitates the development of robust, portable, and accessible analytical tools capable of in situ monitoring.^6,7^ However, a critical materials engineering challenge remains in the fabrication of ubiquitous sensors: conventional electrochemical devices often rely on nonbiodegradable plastic substrates or complex screen-printing processes that suffer from interfacial delamination under mechanical stress.^8–10^ While carbon nanomaterials, particularly graphene, offer exceptional conductivity and surface area,^11^ pristine Graphene Oxide (GO) films are intrinsically brittle, exhibiting low fracture strain (<1%), which limits their standalone application in flexible electronics.^12–14^

To address these mechanical and sustainability limitations, the engineering of functional biocomposites offers a promising pathway. Sodium carboxymethylcellulose (CMC), an abundant cellulose derivative, emerges as an ideal functional matrix.^15^ Beyond its role as a biodegradable binder, CMC interacts with GO via strong hydrogen bonding, effectively preventing the restacking of graphene sheets and conferring essential mechanical flexibility to the composite.^16–18^ Leveraging these properties requires a processing technique that preserves the composite’s integrity while restoring electrical conductivity. Laser Direct Writing (LDW) has revolutionized the field as a mask-free, solvent-free, and scalable lithography technique.^19^ LDW enables the localized reduction of insulating GO into conductive laser-reduced graphene oxide (LrGO) within the biopolymer matrix, thereby promoting the formation of a hierarchical, three-dimensional porous morphology that is highly suitable for electrochemical sensing.^20–26^ This photoreduction process comprises two distinct mechanisms, photochemical and photothermal, with the photothermal pathway predominating when laser irradiation is performed at wavelengths above 390 nm.^27,28^

Despite advances in laser-processed materials, electrochemical monitoring of Paraquat (PQ) – a nonselective herbicide with high acute toxicity – still faces several analytical bottlenecks. A critical challenge is the severe surface passivation of traditional electrodes; during cathodic reduction, intermediate radical cations (PQ^+•^) and neutral species (PQ^0^) form and irreversibly adsorb, fouling the electrode surface.^29,30^ This fouling blocks active sites, significantly compromising signal reproducibility and long-term stability.^31^ Furthermore, while surface functionalization with complex nanomaterials can enhance sensitivity, such architectures often exhibit poor interfacial adhesion and mechanical fragility under repeated flexion, limitations that are particularly detrimental for flexible electronics.^32–34^ Additionally, the typical reliance on liquid cells and labor-intensive sample pretreatment to mitigate matrix effects restricts device miniaturization and precludes noninvasive, point-of-care (PoC) analysis.^35^ Consequently, there is a growing demand for robust, disposable sensing platforms that can withstand surface fouling and enable direct analysis in complex food matrices.

In this work, we report the design and engineering of a freestanding and seamlessly integrated LrGO/CMC platform for noninvasive agrochemical monitoring. By optimizing a scalable casting method followed by precise LDW processing, we developed a flexible sensor in which the active electrode is chemically derived from the support itself, thereby minimizing interfacial failures common in layered devices. The LDW parameters, such as laser power (LP), scan speed (SS), and line interval (LI), were systematically investigated as a core strategy to produce LrGO/CMC electrodes with optimized conductive properties and morphology for pesticide detection. We present a comprehensive characterization of the material’s morphological and electrochemical properties, demonstrating that the optimized Differential Pulse Voltammetry (DPV) technique enables high sensitivity and selectivity for Paraquat (PQ). Finally, expanding the frontiers of wearable agro-electronics, we demonstrate a ″lab-on-a-fruit″ proof-of-concept in which the flexible device is applied directly to orange peels for rapid detection of pesticide residues, offering a resource-efficient solution to modern food safety challenges.

## 2 Results and Discussion

### 2.1 Fabrication and Optimization of LrGO/CMC-Based Devices

We developed a flexible, freestanding Laser-Scribed Graphene (LSG) device applied as an electrochemical sensor. To this end, freestanding films were synthesized by dispersing GO within a CMC biopolymer matrix (Figure 1a). The resulting GO/CMC biocomposite film (Figure 1b) is notably freestanding and scalable (15 × 15 cm), confirming its robustness as an ideal flexible substrate for LDW. This technique is particularly well-suited for this substrate, as the laser energy selectively converts only the surface into conductive LrGO, while the unmodified bulk remains intact. This unmodified bulk serves as an integrated mechanical support for the active LrGO layer, creating a robust monolithic architecture that effectively prevents delamination. Consequently, the final device exhibits a synergistic combination of essential properties: the bulk GO/CMC matrix provides mechanical robustness and flexibility,^15,36^ while the LrGO surface delivers the electrical conductivity and electrochemical surface area required for sensing applications.^36,37^

**Figure 1:**
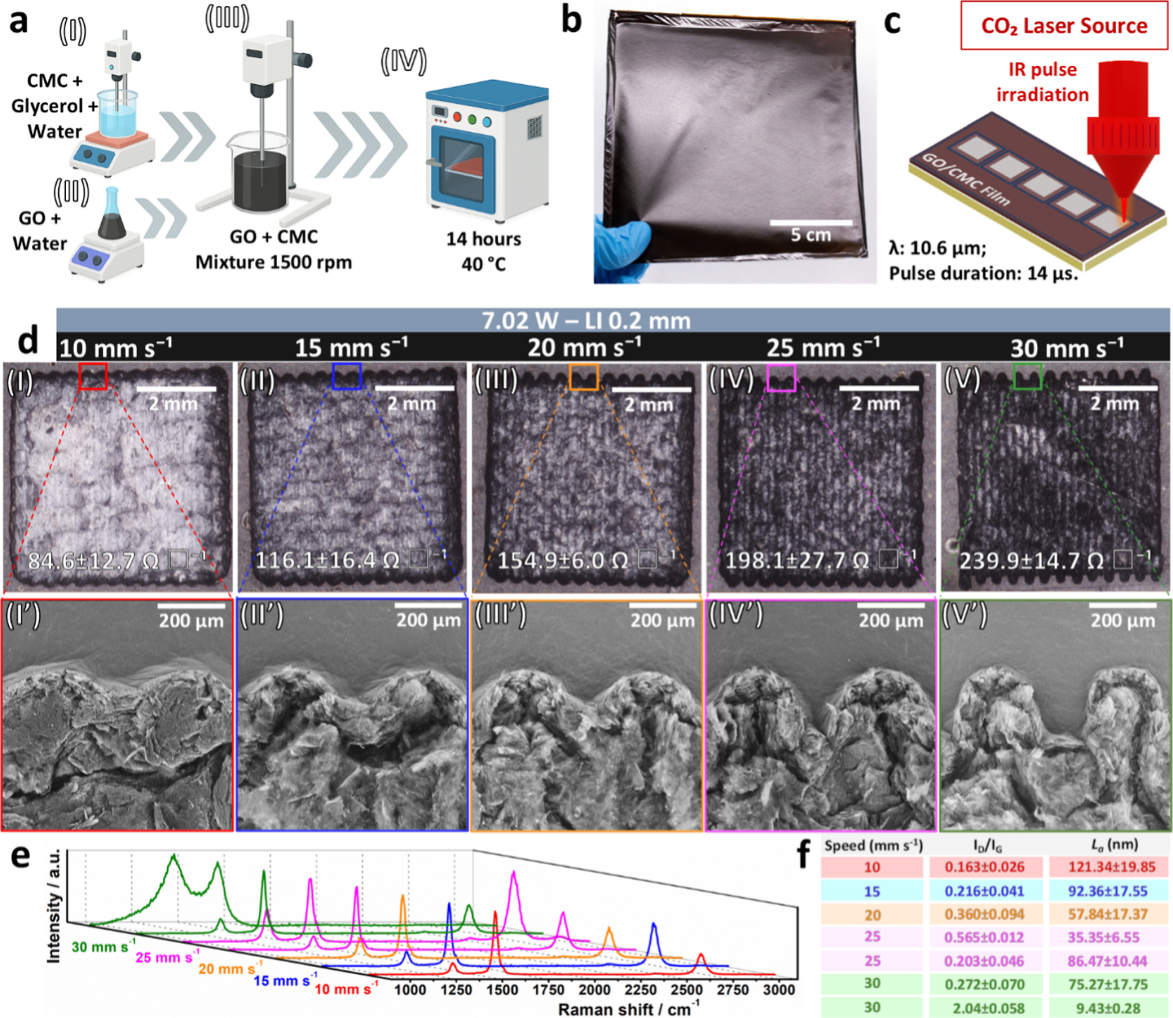
Fabrication and structural characterization of the integrated LrGO/CMC biocomposite platform. (a) Schematic of the solvent-free film synthesis and (b) Digital photograph of the resulting flexible 15 × 15 cm GO/CMC film. (c) Schematic representation of the LDW process using a CO_2_ laser. (d) Scan speed optimization (10–30 mm s^–1^ at 7.02 W and 0.2 mm line interval), displaying digital photographs with respective sheet resistance (*R*_*s*_) values (I–V) and top-view SEM micrographs (I’–V′) of the LrGO-GO/CMC transition borders (sampling sites indicated by colored squares in the digital images). (e) Normalized Raman spectra and (f) I_D_/I_G_ ratios of the LrGO samples, and values of in-plane crystallite size (*L*_*a*_), confirming the efficient restoration of sp^2^ domains at lower speeds.

This method provides a scalable, rapid, and chemical-free route to fabricate LrGO-based devices via an energy-efficient, mask-free photothermal process that simultaneously reduces and patterns GO.^38,39^ For this purpose, a thorough study on the application of the LDW technique was developed, exploring the three main parameters, which were analyzed to identify the ideal engraving condition for the GO/CMC matrix presented in this work: laser power (LP), scan speed (SS), and line interval (LI). The latter, LI, is the spacing between the laser-engraved lines. The structural and morphological characterization of the GO/CMC film, prior to LDW modification, is presented in the Supporting Information (Figure S1). The detailed discussion of both characterizations will be presented in the following sections.

To optimize the infrared laser radiation parameters, we initially engraved 5.0 mm^2^ squares (Figure 1c). The efficacy of the laser-induced reduction was monitored by measuring sheet resistance (Rs), expressed in units of ohms per square (Ω □^–1^). First, the laser power was adjusted (ranging from 6.84 to 7.11 W) at a fixed line interval of 0.1 mm and a scan speed of 20 mm s^–1^.

As shown in Figure S2a, increasing the laser power decreases the sheet resistance of the engraved material. A power threshold of 7.11 W was identified, beyond which complete material ablation occurs. The best electrical properties were achieved at 7.11 W (138.6 Ω □^–1^ ± 20.9) and 7.02 W (152.6 Ω □^–1^ ± 20.5), which showed comparable performance. We selected 7.02 W to ensure greater structural integrity. At 7.11 W, intense photothermal effects caused excessive expansion of graphene sheets and material ejection (Figure S3, IV). These effects, driven by the rapid removal of oxygenated groups and vaporization of interlayer water, generate internal pressure that facilitates ablation.^40^ When excessive, these processes can compromise the mechanical and structural integrity of the LrGO, limiting its practical utility.

Recognizing the critical interplay between power and scan speed for producing stable and reproducible rGO electrodes,^41^ we performed a scan speed study (10–30 mm s^–1^) at the established 7.02 W power and a fixed 0.1 mm line interval (LI) (Figure S2b). Speeds below 10 mm s^–1^ resulted in partial film vaporization due to excessive energy density (Figure S4a, I), hindering characterization. On the other hand, higher speeds reduced the efficiency of the GO-to-LrGO conversion due to insufficient exposure time, yielding poorer electrical properties (Figure S4a­(III–V)). The optimal electrical performance was achieved at 15 mm s^–1^ (97.7 Ω □^–1^ ± 15.9), demonstrating the strong dependence of graphene properties on laser processing conditions (Figure S4a, II). This condition likely represents a balance between exposure time and energy delivery, maximizing the thermal reduction efficiency while preserving structural integrity.^41^

To better understand film vaporization as a function of scanning speed and recording power, the profiles of individual lines printed on GO/CMC films were analyzed using digital images and SEM (Figure S4b), including top-view and cross-sectional images. It is evident that increasing the scanning speed results in narrower tracks (Figure S4b, I–V and I’-V′), with the width varying from 621 to 292 μm. This result already indicates that the line interval (LI) is a fundamental point of study. The cross-sectional images show that at low scan speeds, there is greater material expansion, while at higher speeds (30 mm s^–1^) the laser spot becomes more clearly defined, with limited material ejection along the engraved line.

Beyond LP and SS, these observations indicate that the spacing between engraved lines (Line Interval, LI) also significantly influences the effective laser–material interaction. Although less frequently discussed in the LDW literature, it will be shown here to be fundamental for electrode optimization.

We investigated the effect of varying LI using the optimized power (7.02 W) and scan speed of 10 mm s^–1^. Figure S5a presents a schematic that illustrates how the line interval can be interpreted. Small LI values promote significant overlap between adjacent engraved tracks, leading to (partial) film vaporization due to high energy delivery. On the other hand, large LI results in low line overlap across heterogeneous, partially reduced regions. Increasing the LI from 0.1 to 0.4 improved spatial energy distribution and maintained film integrity. However, the large distance between the LrGO tracks, shown in Figure S5a, III–IV and III–IV’, promotes a higher sheet resistance (Figure S2c) likely due to gaps forming between conduction LrGO pathways, leaving insulating precursor material that compromised device performance. Specifically, a 0.2 mm LI yielded the optimal LrGO tracks with the lowest sheet resistance.

To validate the optimal LDW parameters, a final scan speed optimization was conducted at a power of 7.02 W and a LI of 0.2 mm (Figure S5b). Consistent with previous observations, higher speeds led to inefficient reduction (Figure S5b III–V, III’-V′ and V″), a heterogeneous surface and higher sheet resistance (Figure S2d), whereas speeds below 10 mm s^–1^ caused vaporization. On the other hand, at speeds of 10 and 15 mm s^–1^ (as shown in Figure S5b I–II, I′-II’ and I″), materials exhibiting more organized and deeper LrGO tracks, highlighting the 10 mm^–1^ scan speed, which exhibited the best sheet resistance (84.6 Ω □^–1^ ± 12.7) in consequence of fine-tuning of all parameters discussed above. Finally, the best engraving parameters to obtain an LrGO from a GO/CMC-based freestanding film were observed at 10 mm s^–1^, 7.02 W power, and 0.2 mm line interval.

To elucidate the impact of LDW configurations, we conducted a morphological analysis using digital photographs and scanning electron microscopy (SEM) images acquired at the interface between the laser-treated and untreated regions, complemented by quantitative Raman spectroscopy analysis. The images in Figure 1d demonstrate a systematic evolution of the resulting 3D microstructure, explaining the variations in electrical properties quantitatively supported by the Raman data. The distinct morphological characteristics observed under different processing conditions provide crucial insights into the laser-induced reduction process and its effect on electrode performance.

Digital photographs (Figure 1d, I–V) show a clear macroscopic transition: at lower speeds, etched tracks appear metallic gray and well-defined, whereas at higher speeds they become darker, more opaque, and less uniform. SEM images (Figure 1d, I′-V′) reveal that at lower speeds (higher laser energy density), the formation of deep, interconnected etching lines is promoted, indicating efficient photothermal reduction. The scan speed also dictates the thermal relaxation time during cooling. Faster speeds limit the time available for structural reorganization, leading to the observed changes in surface roughness.^42^ Conversely, at lower speeds, the laser-induced expansion of GO sheets followed by cooling – facilitated by the thermal conductivity of rGO – occurs more slowly, enabling improved structural relaxation and reorganization.^43,44^ These features contrast sharply with the smooth surface of the pristine GO/CMC film before the laser exposure (Figure S1a). Systematic morphological characterization for all conditions is provided in the Supporting Information (Figures S4 and S5).

Structural evolution and graphitic recovery were probed via Raman spectroscopy analysis (Figure 1e). The spectra display the characteristic D band (∼1356 cm^–1^), arising from structural defects and sp^3^ hybridization, and the G band (∼1589 cm^–1^), corresponding to the first-order scattering of the E_2g_ mode in sp^2^ carbon domains. The 2D band (∼2700 cm^–1^) further elucidates the stacking order.^45^ Importantly, the intensity ratio of the D and G bands (I_D_/I_G_) serves as a quantitative metric for the degree of disorder and, inversely, the in-plane crystallite size (*L*_*a*_).^46^

Deconvolution of the spectra (Figure S6) reveals a sharp contrast between the pristine GO/CMC precursor (Figure S1b) and the laser-treated regions. A clear inverse correlation exists between the laser scan speed (energy density) and the structural defect density (Figure 1f). At lower speeds (10–20 mm s^–1^), the prolonged laser interaction provides sufficient thermal energy not only for deoxygenation but also for thermodynamic reorganization of the carbon lattice.^47^ Notably, the optimal condition of 10 mm s^–1^ yielded the lowest I_D_/I_G_ ratio of 0.163. According to the Tuinstra-Koenig relation^46,48^ (calculation details in Note S1), this minimized ratio corresponds to a significant expansion of the *L*_*a*_ to approximately 121.34 nm. This value is markedly higher than those observed for faster scanning speeds, confirming the effective “healing” of vacancy defects inherent to the GO precursor into large, ordered sp^2^ domains.^19,49–51^

In contrast, transient thermal exposure at higher speeds (25–30 mm s^–1^) induced a kinetic instability, resulting in drastically reduced crystallite sizes ranging from 9.43 to 35.35 nm. The resulting spectra exhibited substantial inhomogeneity, necessitating their segregation into distinct groups. This spectral variance reflects microscopic spatial heterogeneity, where localized regions failed to reach the activation energy required for complete graphitization, leaving a mosaic of reduced and partially oxidized domains – a heterogeneity also visually apparent in the macrographs (Figure 1d, I–V). This structural disorder acts as a barrier to charge transport, directly correlating the high/variable I_D_/I_G_ ratios (and consequently small *L*_*a*_) with the inferior electrical conductivity observed. Thus, precise modulation of the scanning speed is identified as the governing factor in transitioning from a disordered oxide to a high-performance conductive electrode.

### 2.2 Electrochemical Device Assembly and Characterization

To translate the optimized nanocomposite into a functional analytical platform, a miniaturized three-electrode system was fabricated directly onto the freestanding GO/CMC film using the previously optimized LDW parameters (7.02 W, 10 mm s^–1^, 0.2 mm LI) to ensure maximum conductivity and structural quality, as shown in Figure 2a, I–II. The resulting architecture comprises a working electrode (WE), a counter electrode (CE), and a pseudoreference electrode (RE). To ensure stable potential and efficient charge collection, silver ink was applied to the contact pads and the pseudoreference track (Figure 2a, III). Subsequently, the nonactive areas were passivated with an insulating layer to precisely define the geometric working area and prevent electrolyte leakage (Figure 2a, IV). The detailed layout and dimensions are provided in Figure 2b.

**Figure 2:**
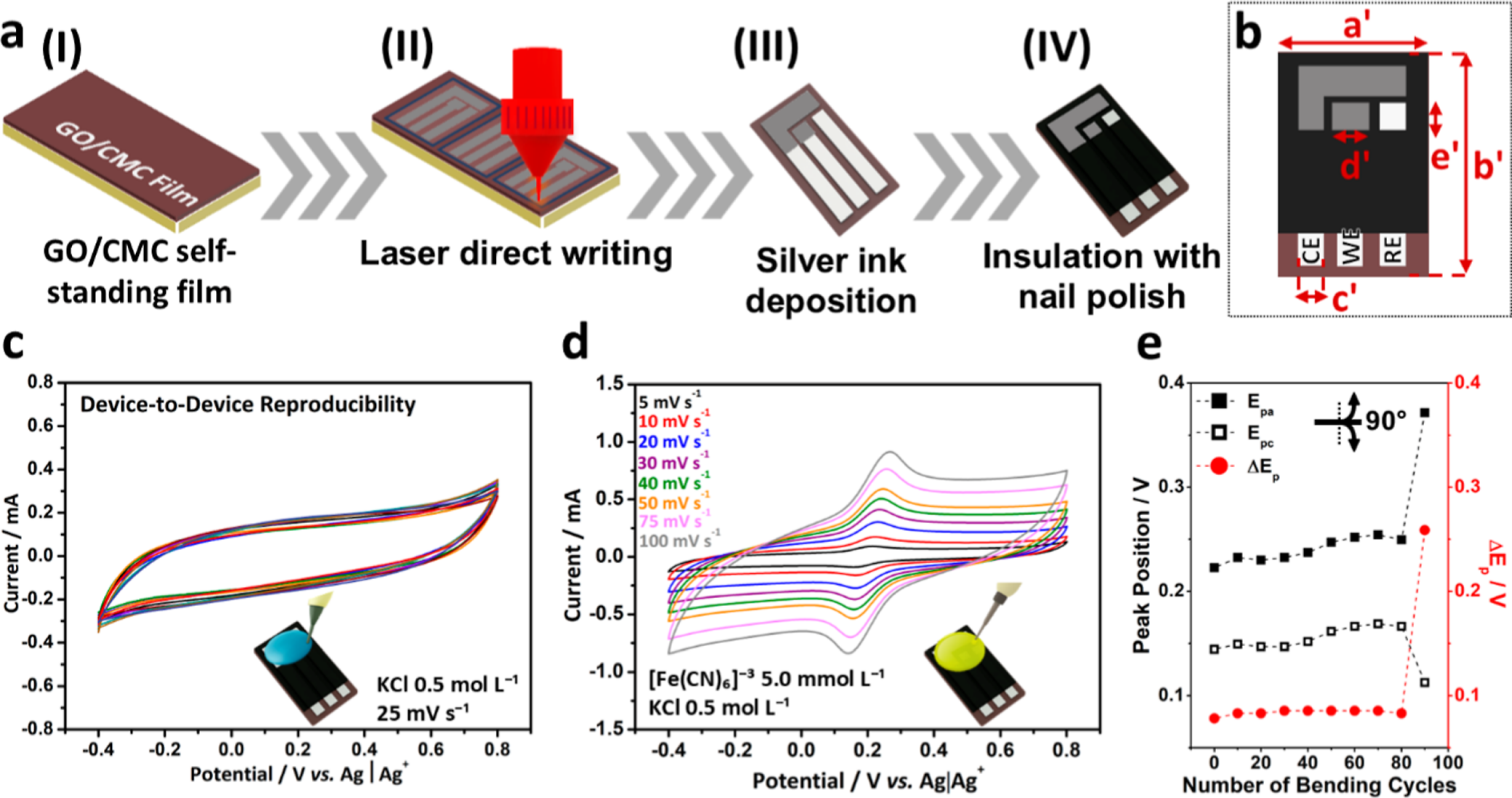
Integrated fabrication, geometric design, and electrochemical consistency of the LrGO/CMC platform. (a) Fabrication steps of the miniaturized sensor array, including laser engraving, silver ink deposition, and passivation. (b) Schematic layout of the three-electrode system with optimized dimensions: a’ = 14.9 mm, b’ = 27.2 mm, c’ = 2.1 mm, d’ = 3.7 mm, and e’ = 3.3 mm. The resulting geometric area of the working electrode (WE) is 0.12 cm^2^. (c) Device-to-device reproducibility (*n* = 10) assessed via Cyclic Voltammetry (CV) in 0.5 mol L^–1^ KCl (25 mV s^–1^, microdroplet configuration). (d) CV profiles in 5.0 mmol L^–1^ microdroplet of [Fe­(CN)_6_]^3‑/4‑^ at varying scan rates (5 to 100 mV s^–1^), demonstrating reversible kinetics. (e) Stability of the peak-to-peak potential separation (Δ*E*_*p*_) over 90 bending cycles at 90°, confirming the structural integrity of the integrated architecture.

All electrochemical measurements related to the characterization of the WE surface were performed by adding 150 μL of solution onto the active area (inset in Figure 2c,d). Electrochemical consistency of the fabrication process was evaluated by assessing device-to-device reproducibility (n = 10) using cyclic voltammetry (CV) in a 0.5 mol L^–1^ KCl electrolyte (Figure 2c). The sensors exhibited a uniform capacitive current density, with a Relative Standard Deviation (RSD) of 11.7%, confirming the method’s scalability for disposable screening devices. Additional electrolyte compatibility studies are shown in Figure S7a.

Electron transfer kinetics were evaluated using the [Fe­(CN)_6_]^3–^/Fe­(CN)_6_]^4–^ redox probe (Figure 2d). The electrodes exhibited well-defined reversible behavior, with a peak-to-peak potential separation (Δ*E_p_*) of 44 mV at a scan rate of 5 mV s^–1^. An Δ*E*_p_ value below the theoretical Nernstian limit of 59 mV indicates that the electrochemical response approaches that of an ideal reversible thin-layer system. In this regime, mass transport limitations are significantly reduced due to the confinement of the electroactive species within the porous electrode architecture, while the highly efficient charge transfer is attributed to the interconnected porous and rough structure of the LrGO/CMC electrodes. The linear relationship between the peak current (*I*_*p*_) and the square root of the scan rate (*v* ^1/2^) (Figure S7b, *R* ^2^ > 0.99) confirms a diffusion-controlled process.

To evaluate the accessible surface area and structural porosity, the electrochemically active surface area (ECSA) was determined via CV in the non-Faradaic region using 0.5 M KCl. The CV profiles obtained at the scan rates ranging from 10 to 100 mV s^–1^ are presented in Figure S7c. The Double-layer Capacitance (*C*_*dl*_) method was selected as the most representative for our 3D platform, rather than faradaic probes (such as the Randles-Sevcik equation). This choice is justified because faradaic models often overestimate the active area in hierarchical porous networks due to radial diffusion fluxes at structural boundaries and edge effects from laser-etched patterns. In contrast, the capacitive response provides a more robust and thermodynamically accurate measurement of the truly wetted electrode–electrolyte interface.^52–54^ A linear relationship was observed when plotting capacitive current density (*j*) versus scan rate (*v*) (Figure S7d). The slope of this regression corresponds to the area-specific double-layer capacitance (*C*_*s*_), which allows the calculation of the ECSA according to eqs 1–3.^26,55^

1 $$j=\frac{I_{a}}{S_{geom}}$$

2 $$C_{s}=\frac{j}{v}$$

3 $$ECSA=\frac{S_{geom}\times C_{s}}{C_{ref}}$$

Here, *j* represents the current density, *I*_*a*_ is the measured anodic current, *v* is the scan rate, and *S*_geom_ is the geometric area of the electrode (0.12 cm^2^). The term *C*_ref_ denotes the specific double-layer capacitance of Laser-Induced Graphene (LIG), which exhibits electroactive behavior similar to that of LrGO, and is set to 3.9 mF cm^–2^.^56^ The average electrode area obtained from three replicates was 0.274 ± 0.072 cm^2^, representing slightly more than twice the geometric area. This enhancement is attributed to the laser-induced morphological expansion observed in SEM, which results in a hierarchical, rough LrGO/CMC network that maximizes the electrode–electrolyte interface.

Fundamentally, the practical viability of wearable electronics hinges on mechanical robustness. The sensor was subjected to a dynamic stress test comprising 90 continuous bending/unbending cycles at 90°, with the electrochemical response of the redox probe monitored. As shown in Figure 2e, the device exhibited good stability, with an Δ*E*_*p*_ variation of only 6.24% even after extensive cycling. This robustness validates the structural reinforcement provided by the CMC biopolymer matrix.^57–59^ Consistent with the literature, the strong hydrogen bonding network facilitates efficient stress transfer across the composite interface, preventing microcrack propagation.^60–62^ Unlike conventional screen-printed electronics, which are prone to interfacial delamination due to mechanical mismatch,^9,10^ our integrated architecture ensures that the conductive path remains intrinsic to the flexible substrate, preserving kinetic integrity under physical deformation. Further visual details of the mechanical testing are shown in Video 1.

### 2.3 Analytical Performance: Electrochemical Detection of Paraquat (PQ)

The electrochemical performance of the LrGO device toward Paraquat detection was evaluated after optimizing the Differential Pulse Voltammetry (DPV) conditions to establish ideal operational parameters. The supporting electrolyte optimization was assessed by varying pH values, as shown in Figure 3a, using a 625.0 μmol L^–1^ PQ solution. The resulting voltammograms, recorded in an anodic scan direction, revealed a broad oxidation peak at approximately −0.61 V. This signal is attributed to the oxidation of the reduced paraquat specie,^30^ schematically illustrated in Figure 3b.

**Figure 3:**
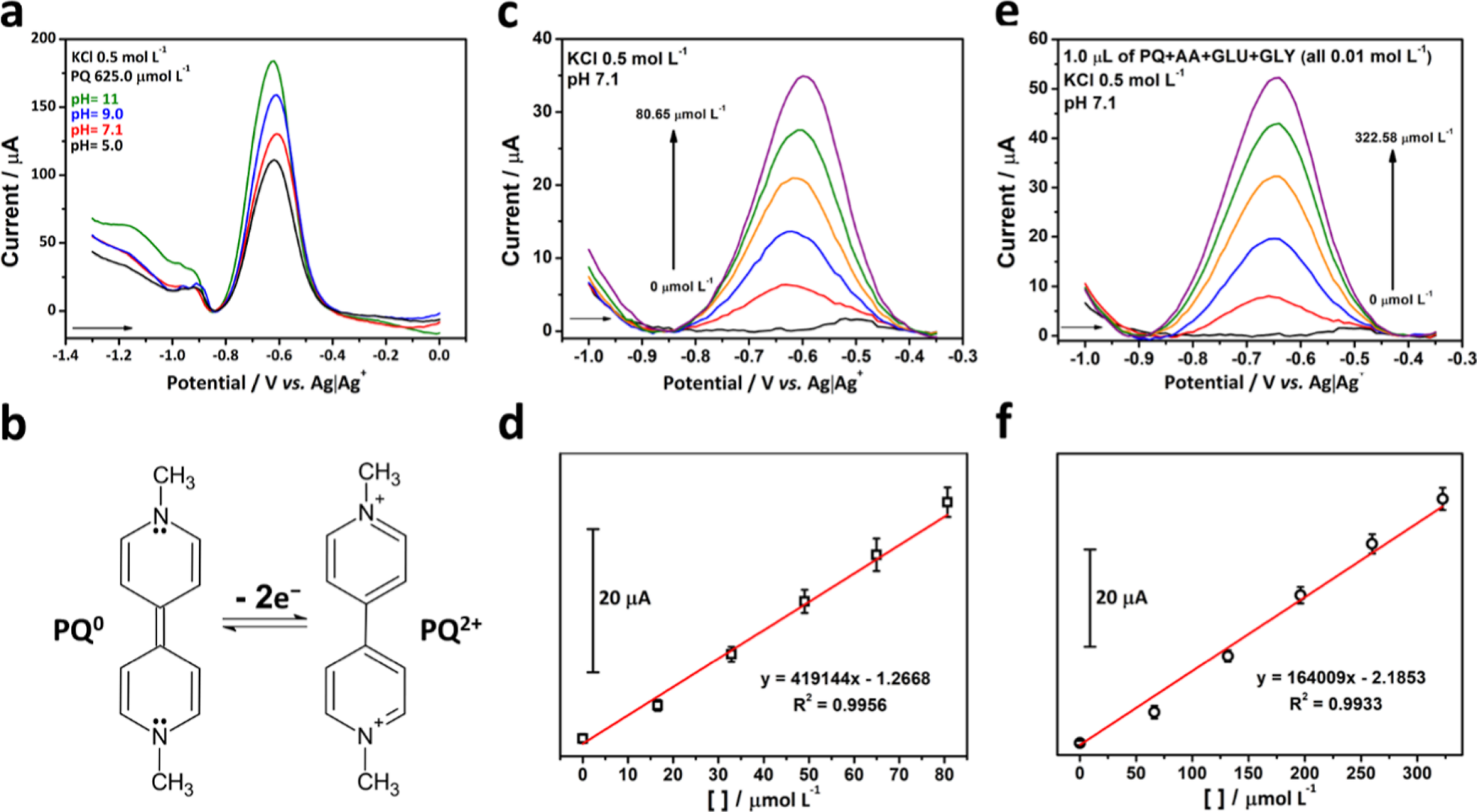
Analytical optimization and sensing performance of the LrGO/CMC platform for Paraquat (PQ) detection. (a) Influence of electrolyte pH (5.0–11.0 in 0.5 mol L^–1^ KCl) on the response to 625 μmol L^–1^ PQ. (b) Schematic of the reversible PQ^0^/PQ^2+^ redox mechanism. (c) Differential pulse voltammetry (DPV) curves for successive PQ additions (16.13–80.65 μmol L^–1^ and (d) the corresponding linear calibration plot, yielding an LOD of 5.7 μmol L^–1^. (e) Selectivity assessment against common agricultural interferents (ascorbic acid, glucose, and glyphosate). (f) Matrix-matched calibration curve demonstrating the sensor’s quantitative robustness (LOD matrix = 7.8 μmol L^–1^.

A clear pH-dependence was observed. Higher current intensities were observed in alkaline media (pH 9.0 and 11.0), accompanied by a sharp shift in potential. The current enhancement at elevated pH is attributed to the deprotonation of the remaining COO^–^ groups on the LrGO/CMC composite surface. This increases the negative surface charge, enhancing the electrostatic preconcentration of the PQ^2+^ cation at the electrode interface before the anodic scan.^63^ However, despite the higher signal, these alkaline conditions resulted in poor signal stability and reproducibility, as detailed in Figure S8a (consecutive scans) and Figure Sb (*I*_*p*_ and standard deviation). In contrast, pH 5.0 and 7.1 yielded comparable signals. Therefore, pH 7.1 was selected as the optimal condition. This choice offers a dual advantage: it ensures compatibility with real agricultural samples, simplifying on-site analysis,^52^ while simultaneously preserving the structural integrity of the CMC biopolymer matrix against excessive swelling or hydrolysis, often induced by extreme pH media.^64^

Electrochemical sensing of PQ was conducted under optimized DPV conditions (scan range: −1.0 to −0.35 V; step height: 10 mV; pulse amplitude: 75 mV; pulse width: 20 ms) in a microdroplet configuration. For this, a 150.0 μL drop of the optimized electrolyte (KCl 0.5 mol L^–1^, pH 7.1) was dispensed onto the LrGO electrode system, followed by successive additions of 1.0 μL of aqueous PQ solution (2.5 × 10^–3^ mol L^–1^). As depicted in Figure 3c, the representative differential-pulse voltammograms reveal a distinct enhancement in the anodic peak current proportional to the analyte concentration within the range of 16.13–80.65 μmol L^–1^. The corresponding calibration isotherm (Figure 3d) correlates the oxidation peak current (*I*_*p*_) to PQ concentration and demonstrates excellent linearity over this range (*R* ^2^ = 0.9956). From this linear regression, the sensitivity (*S*) – corresponding to the slope – and the standard deviation of the blank (*s*) were used to calculate the limit of detection (LOD) and limit of quantification (LOQ), according to eqs 4 and 5

4 $$LOD=\frac{3s}{S}$$

5 $$LOQ=\frac{10s}{S}$$

Based on these parameters (n = 3 blank measurements), the sensor yielded an LOD of 5.7 ± 1.2 μmol L^–1^ (1.06 ± 0.221 mg L^–1^) and an LOQ of 18.7 ± 4.1 μmol L^–1^ (3.48 ± 0.76 mg L^–1^). To evaluate the precision of the method, the RSD was calculated for the detection of 49.02 μmol L^–1^ of PQ (from analytical curve replicates), yielding 5.16% (n = 3), demonstrating adequate repeatability for quantitative analysis.

From a toxicological perspective, although the LOD (≈1.06 mg L^–1^) is above the stringent drinking water limits established by agencies such as the EPA (0.003 mg L^–1^),^55^ it remains highly relevant for detecting acute contamination scenarios. Considering that the estimated lethal dose of PQ for humans is approximately 35 mg kg^–1^ ^65^, the ingestion of small volumes of concentrated solutions is fatal. Therefore, the developed sensor provides sufficient sensitivity for rapid on-site screening in agricultural applications.

To evaluate the sensor’s selectivity, the electrochemical response was assessed in the presence of coexisting species typically found in fruits (ascorbic acid – AA; glucose – GLU) and another agricultural pesticide (glyphosate – GLY). Figure 3e demonstrates that the addition of a complex mixture containing equimolar concentrations of PQ, AA, GLU, and GLY resulted in negligible signal suppression, confirming the devicés specificity toward Paraquat.

This selectivity was further quantified through a targeted interference study (Figure S9), comparing the pure PQ signal against multicomponent mixtures. The results showed signal variation of only 4.72% in the presence of AA, H_2_O_2_, mancozeb, and 5.54% for the quaternary mixture (PQ/AA/GLU/GLY), values within acceptable analytical limits. To rigorously assess performance in complex media, a matrix-matched calibration curve was constructed (Figure 3f). Under these conditions, the device maintained its quantitative capability, exhibiting a LOD of 7.8 μmol L^–1^ (1.45 mg L^–1^) and a LOQ of 26 μmol L^–1^ (4.84 mg L^–1^). Although slightly higher than in pure electrolyte, this minimal deviation highlights the robustness of the LrGO/CMC architecture, demonstrating its capability to maintain reliable signal transduction even within a complex, multicomponent environment.

Finally, the accuracy of the proposed LrGO/CMC platform was validated by comparing its performance with High-Performance Liquid Chromatography (HPLC). The HPLC analytical curve parameters were used to calculate the theoretical limits using the standard deviation of residuals of calibration samples,^66^ resulting in an LOD of 4.63 μmol L^–1^ (0.86 mg L^–1^) and an LOQ of 14.04 μmol L^–1^ (2.61 mg L^–1^). The results obtained with the electrochemical platform showed excellent correlation with the standard chromatographic method, confirming the accuracy and reliability of the freestanding electrodes for Paraquat screening. Detailed HPLC curves are available in Figure S10.

### 2.4 ″Lab-On-a-Fruit″ Proof-of-Concept for In Situ Monitoring

Finally, to demonstrate the potential of the LrGO platform for precision agriculture, we implemented a “lab-on-a-fruit” proof-of-concept for noninvasive and rapid detection of PQ directly on fruit surfaces. The coupling of flexible sensing architectures with portable electrochemical instrumentation represents a promising strategy to modernize food safety protocols. To this end, we conducted a simulated screening using oranges: a control group (pesticide-free) and a group intentionally contaminated with PQ (100.0 μL, 1.0 × 10^–2^ mol L^–1^) as depicted in Figure 4a (I–II).

**Figure 4:**
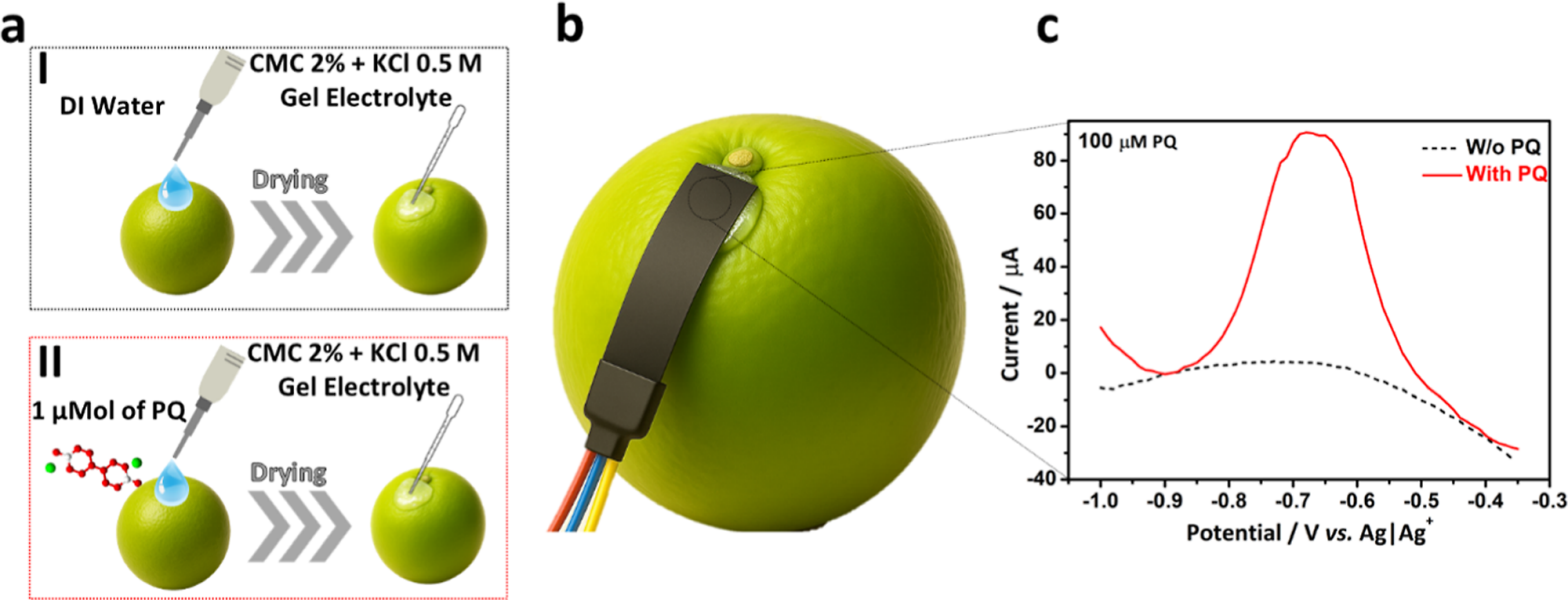
″Lab-on-a-fruit″ proof-of-concept for noninvasive in situ agrochemical monitoring. (a) Schematic of the detection strategy and sample preparation for both control and PQ-contaminated (1.0 × 10^–2^ mol L^–1^) orange peels using a CMC-gel electrolyte. (b) In situ experimental setup illustrating the flexible LrGO sensor conformally positioned over the fruit surface. (c) Comparative DPV responses, displaying the PQ oxidation peak at ≈ −0.61 V for the contaminated sample (red line) versus the pesticide-free control (black line).

The interface is decisive for this noninvasive approach. We employed a CMC-based hydrogel electrolyte (2% w/v CMC in 0.5 mol L^–1^ KCl) applied directly to the fruit skin. This gel serves a dual function: it acts as an ionic bridge and as a nondestructive extraction medium. Gentle friction was applied to the surface to facilitate the diffusion of orange peel residues into the gel matrix. The flexible LrGO device was then conformally positioned over the treated area, establishing the LrGO/Gel/Fruit electrochemical interface (Figure 4b).

The sensing performance is shown in Figure 4c. A distinct oxidation peak at −0.61 V was observed for the contaminated sample, clearly indicating the presence of the herbicide. Indeed, the control sample yielded no discernible electrochemical signature within the potential window, validating the method’s specificity. Notably, the entire screening workflow – from gel application to signal acquisition via a portable potentiostat – was completed in under 1 min (30 s for extraction +30 s for analysis, as demonstrated in Video 2). This “sample-to-answer” speed, combined with the device’s robust flexibility, confirms the suitability of the LrGO/CMC platform as a next-generation wearable tool for rapid, in situ agrochemical monitoring.

## 3 Conclusion

In summary, we have successfully engineered a scalable, freestanding electrochemical platform based on LrGO/CMC biocomposites. By leveraging a solvent-free Laser Direct Writing process, we achieved a highly conductive (84.6 Ω □^–1^) and seamless architecture where the active electrode is intrinsically derived from the supporting matrix. This design effectively eliminates interfacial delamination, a common issue in printed electronics, ensuring exceptional mechanical integrity and stable electron-transfer kinetics even under dynamic bending stress. Analytically, the sensor combines high sensitivity (LOD of 5.7 ± 1.2 μmol L^–1^ and an LOQ of 18.7 ± 4.1 μmol L^–1^) with selectivity against complex agricultural matrices. Validated via a ″lab-on-a-fruit″ proof-of-concept, the platform enabled rapid (<1 min), noninvasive detection of pesticide residues directly on fruit surfaces. These findings bridge the gap between functional materials engineering and precision agriculture, offering a sustainable and robust approach to next-generation wearable agro-electronics.

## 4 Methods

### 4.1 Materials

Graphite powder (Grafine) was supplied by Nacional de Grafite (Brazil). Sodium Carboxymethylcellulose (CMC, medium viscosity) and Glucose (C_6_H_12_O_6_) were purchased from Êxodo Científica. Paraquat dichloride hydrate (PESTANAL, C_12_H_14_Cl_2_N_2_.*x*H_2_O), Mancozeb (PESTANAL, C_8_H_12_MnN_4_S_8_Zn), Potassium chloride (KCl), Sulfuric acid (H_2_SO_4_), Potassium persulfate (K_2_S_2_O_8_), Phosphorus pentoxide (P_2_O_5_), Potassium permanganate (KMnO_4_), Hydrogen peroxide (H_2_O_2_), and conductive silver ink (SunTronic) were obtained from Sigma-Aldrich. Hydrochloric acid (HCl), Acetone (C_3_H_6_O), Potassium ferricyanide (K_3_[Fe­(CN)_6_]), and Ascorbic acid (C_6_H_8_O_6_) were acquired from Neon Commercial. Glycerol was obtained from CRQ. Commercial nail polish (Risque, Coty Inc.) was used as a dielectric layer. All solutions were prepared using deionized water (resistivity >0.05 μΩ cm).

### 4.2 Synthesis of Graphene Oxide

Graphene Oxide (GO) was synthesized via a modified Hummers method, as previously reported by our group.^67–69^ Briefly, graphite powder underwent a preoxidation step using a mixture of K_2_S_2_O_8_ and P_2_O_5_ dissolved in concentrated H_2_SO_4_ at 80 °C. The preoxidized graphite was subsequently oxidized by the addition of KMnO_4_ in an ice bath (0 °C). Then, H_2_O_2_ was added to the mixture to terminate the reaction. The resulting dispersion was purified with 3.4% HCl, filtered, and washed with acetone.

### 4.3 Preparation of Freestanding GO/CMC Biocomposites

The freestanding GO/CMC films were fabricated via solution casting. Initially, 600 mg of GO were dispersed in 70 mL of deionized water (hydration for 60 min, magnetic stirring for 30 min, and ultrasonication for 30 min). Simultaneously, a 1% (w v^–1^) CMC solution containing glycerol as a plasticizer (20% w w^–1^ relative to the polymer mass) was prepared under mechanical stirring at 1000 rpm (60 min, 75 ± 5 °C). Subsequently, 30 g of the CMC solution was incorporated into the GO dispersion (yielding a final 2:1 GO/CMC mass ratio). The mixture was stirred (1500 rpm, 60 min), poured onto acrylic substrates (15 × 15 cm), and dried in an air-circulation oven at 40 °C for 14 h.

### 4.4 Preparation of CMC-Based Hydrogel Electrolyte

The gel electrolyte was prepared by dissolving CMC at a concentration of 2% (w v^–1^) in a previously prepared 0.5 mol L^–1^ KCl aqueous solution. The mixture was stirred continuously (1000 rpm) at room temperature for 2 h until a viscous, homogeneous hydrogel was obtained.

### 4.5 Preparation of LrGO Electrodes via LDW

The sensing platforms were fabricated via Laser Direct Writing (LDW) using a CO_2_ laser cutter (Work Special Laser WS 6040; λ = 10.6 μm, pulse width ≈14 μs, max power 90 W). The optimized parameters for obtaining integrated electrodes were a power of 7.02 W (7.8% of max power), a scan speed of 10 mm s^–1^, and a focal distance of 9.0 mm in an ambient atmosphere. The geometry of the three-electrode system was designed using LaserCAD software (V8.15.15, TROCEN).

### 4.6 Electrochemical Measurements and HPLC analysis

All electrochemical assays were conducted using an EmStat4s potentiostat (PalmSens) controlled by PSTrace 5.9 software. Measurements were performed using a microdroplet configuration (150 μL of electrolyte). Cyclic Voltammetry (CV) was conducted using a redox probe (5.0 mmol L^–1^) in 0.5 mol L^–1^ KCl, with scan rates ranging from 5 to 100 mV s^–1^. Paraquat detection was performed using Differential Pulse Voltammetry (DPV) in 0.5 mol L^–1^ KCl (pH 7.1). The optimized DPV protocol included an electrochemical conditioning step at −1.0 V for 7 s, followed by an anodic scan from −1.0 to −0.35 V with a step height of 10 mV, pulse amplitude of 75 mV, and pulse width of 20 ms. Selectivity assays were performed against solutions of glucose, ascorbic acid, hydrogen peroxide, mancozeb, and glyphosate (1.0 × 10^–2^ mol L^–1^) under identical conditions. For the comparative validation study, PQ was analyzed by HPLC using a mobile phase of 0.01% (v/v) aqueous acetic acid and acetonitrile (95:5, v/v). The flow rate was maintained at 1.0 mL min^–1^, with an injection volume of 50 μL. The column temperature was controlled at 30 °C, and the detection wavelength was set at 257 nm.^33^ The analytical limits (LOD and LOQ) were established following the EURL guidance based on the standard deviation of the intercept and the slope of the calibration curve.^66^

### 4.7 “Lab-On-a-Fruit” Proof-of-Concept Assay

The in situ detection capability was evaluated using fresh oranges (*Citrus sinensis*) purchased from a local market. Prior to the assay, the fruits were washed with deionized water and dried at room temperature. The samples were divided into two groups: Control Group, treated with 100 μL of deionized water; and Contaminated Group, intentionally spiked with 100 μL of a Paraquat solution (1.0 × 10^–2^ mol L^–1^) applied directly to the fruit peel. The droplets were allowed to dry thoroughly under ambient conditions. Subsequently, a small aliquot of CMC-based hydrogel electrolyte was applied to the target area and gently rubbed for 30 s to facilitate analyte extraction. The flexible LrGO device was then positioned conformally over the gel-coated area, and DPV measurements were performed using a portable EmStat4s potentiostat under the optimized parameters described above.

## 5 Material Characterization

Laser parameter optimization was monitored via sheet resistance (*R*_*s*_) measurements using an Ossila Four-Point Probe System (model T2001A4, Ossila Ltd., Sheffield, UK) equipped with a probe head featuring 1.27 mm spacing. Measurements were conducted at room temperature, and specific geometric correction factors for the laser-etched patterns were automatically applied via the Ossila software to ensure technical accuracy. Surface morphology was analyzed by Scanning Electron Microscopy (SEM, PHENOM PROX, PhenomWorld). Raman spectra were acquired using a confocal Raman microscope (objective of 50× magnification) (Alpha 300R, WITEC) operating at an excitation wavelength of 532 nm, with a power of 1.5 mW. The spectra were acquired with an integration time of 5 s and a total of 10 spectra accumulated.

## Supplementary Material







## Notes

Morphological and structural characterization (SEM and Raman) of the precursor GO/CMC film; systematic optimization of laser power, scan speed, and line interval (LI) parameters; comprehensive optical and SEM micrographs of laser-etched tracks; deconvoluted Raman spectra for crystallite size estimation; electrochemical assessment of supporting electrolytes; linear correlations for redox kinetics and ECSA calculation; signal stability and reproducibility evaluation at various pH levels; and detailed selectivity profiles against complex interfering mixtures (PDF)
Demonstration of the mechanical robustness and flexibility of the integrated LrGO/CMC electrodes during dynamic stress tests (MP4)
Real-time “lab-on-a-fruit” screening workflow, illustrating the rapid extraction and noninvasive detection of paraquat on orange peels (MP4)

## Abbreviations

LDW: laser direct writing
LrGO: laser-reduced graphene oxide
CMC: sodium carboxymethylcellulose
GO: graphene oxide;
DPV: differential pulse voltammetry
PQ: paraquat
LOD: limit of detection
LOQ: limit of quantification
CV: cyclic voltammetry
ECSA: electrochemically active surface area
SEM: scanning electron microscopy
LI: line interval
WE: working electrode
CE: counter electrode
RE: reference electrode
AA: ascorbic acid
GLU: glucose
GLY: glyphosate
RSD: relative standard deviation.
