# Supplementary material for: Laser- Written Freestanding Electrodes Based on Laser-Reduced Graphene Oxide/Carboxymethylcellulose for Non-Invasive On-Food Paraquat Detection
Source: ACS Omega. 2026 Jul 17;11(30):45146–56. doi: 10.1021/acsomega.6c03514 (PMC13448938; doi:10.1021/acsomega.6c03514)
Supplement: Supplementary file 1 [file ao6c03514_si_001.pdf]

SUPPORTING INFORMATION

Laser- Written Freestanding Electrodes Based on Laser-Reduced Graphene Oxide/Carboxymethylcellulose for Non-Invasive On-Food Paraquat Detection

*Vinicius N. B. Souza[1], Vitor H.N. Martins[2], Maria H. Verdan[2], Cecilia C. C. Silva[3,4], Silvia M. Martelli[1], and Victor H.R. Souza[2]**

[1] Faculty of Engineering, Universidade Federal da Grande Dourados (UFGD), Dourados, MS, 79804-970, Brazil.

[2] Faculty of Exact Science and Technology, Universidade Federal da Grande Dourados (UFGD), Dourados, MS, 79804-970, Brazil.

[3] MackGraphe, Mackenzie Institute for Research in Graphene and Nanotechnologies, Mackenzie Presbyterian Institute, São Paulo, SP, 01302-907, Brazil.

[4] School of Engineering, Mackenzie Presbyterian University, São Paulo, SP, 01302-907, Brazil.

Laser direct writing; Biocomposites; Electrochemical sensors; Flexible electronics; Food safety.

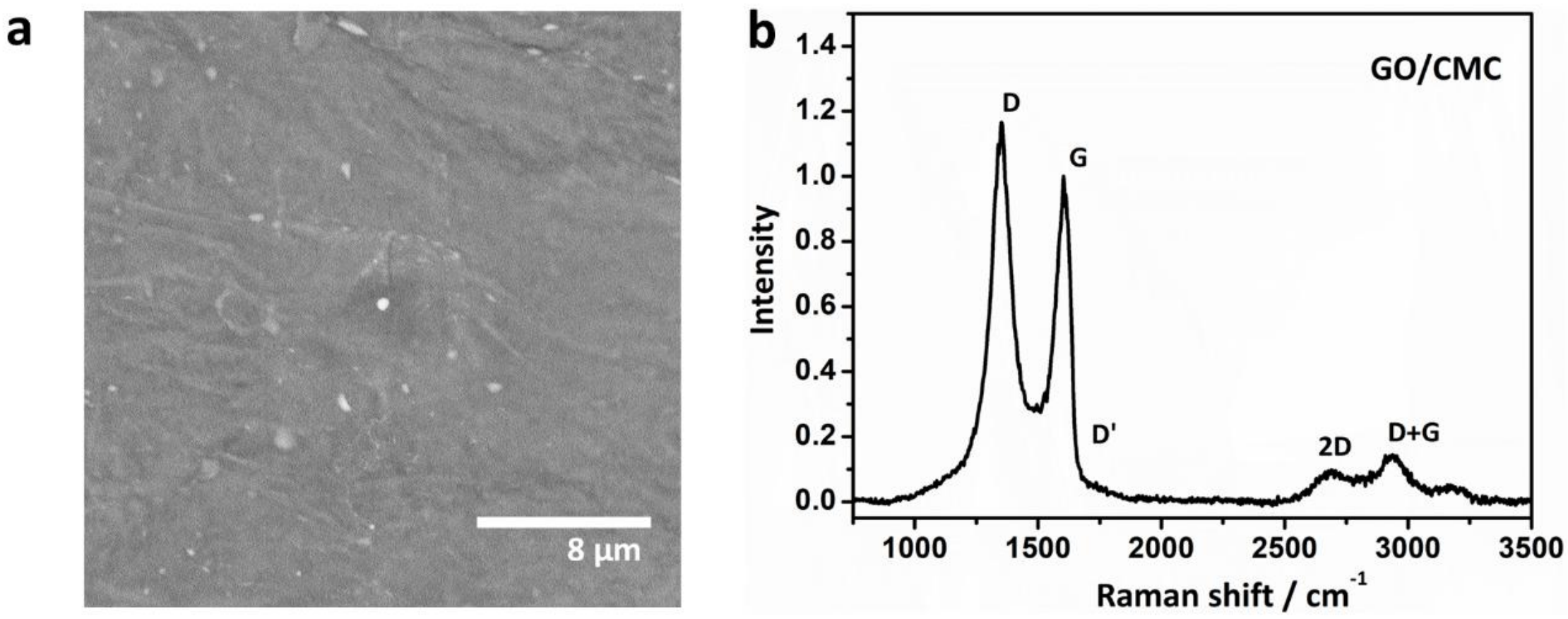

Figure S1. Morphological and structural characterization of the pristine GO/CMC bio-composite film before laser processing. **(a)** Scanning Electron Microscopy (SEM) micrograph of the film surface. **(b)** Raman spectrum of the precursor material.

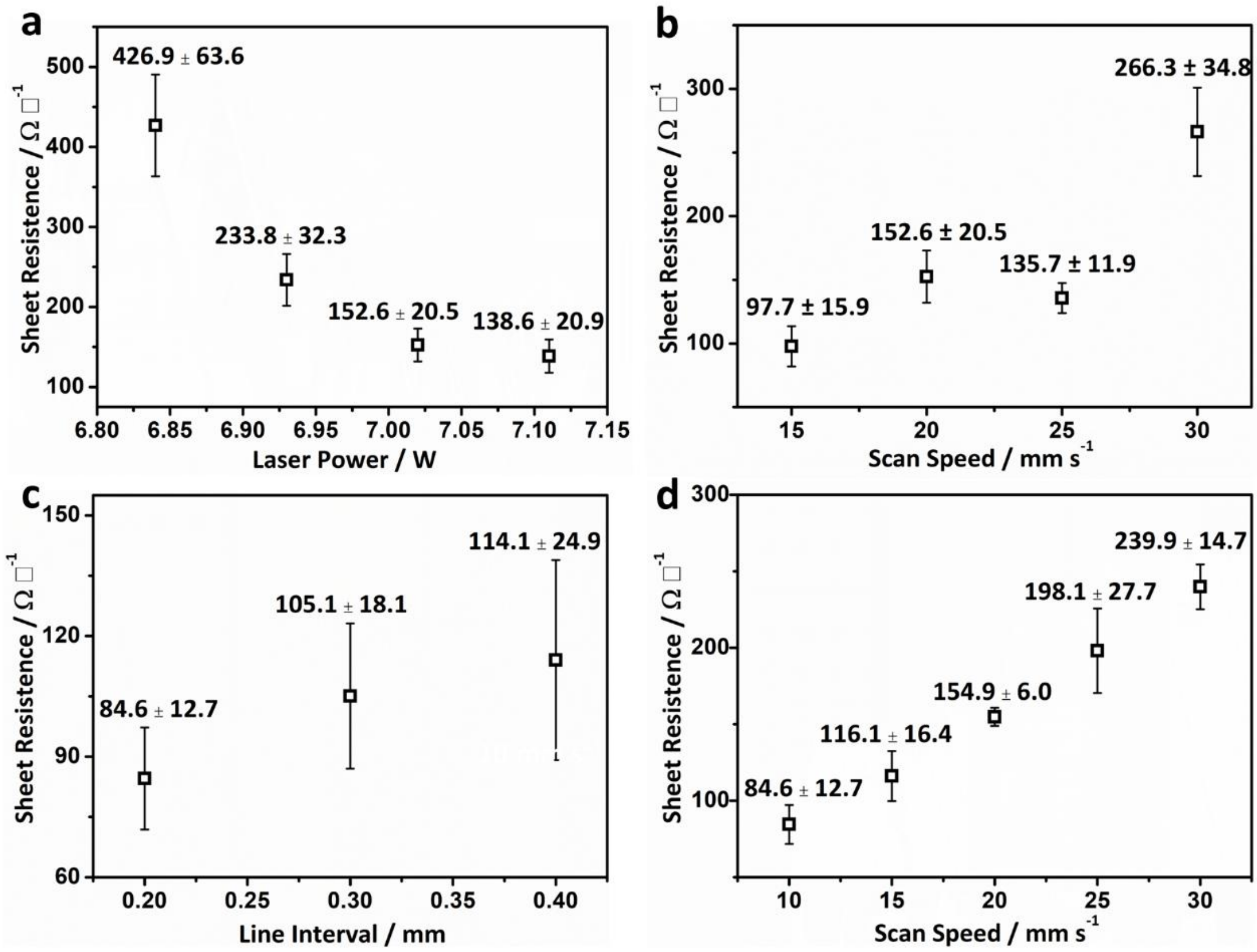

Figure S2. Systematic optimization of LDW parameters on LrGO/CMC squares (5 x 5 mm). **(a)** Effect of laser power variation (6.84 – 7.11 W) at a fixed SS of 20 mm s^{-1} and LI of 0.1 mm. **(b)** Effect of SS (15 – 30 mm s^{-1}) at a fixed LP of 7.02 W and LI of 0.1 mm. **(c)** Influence of LI variation at a fixed LP of 7.02 W and SS of 10 mm s^{-1}. **(d)** Final SS optimization using pre-selected parameters (7.02 W and 0.2 mm LI).

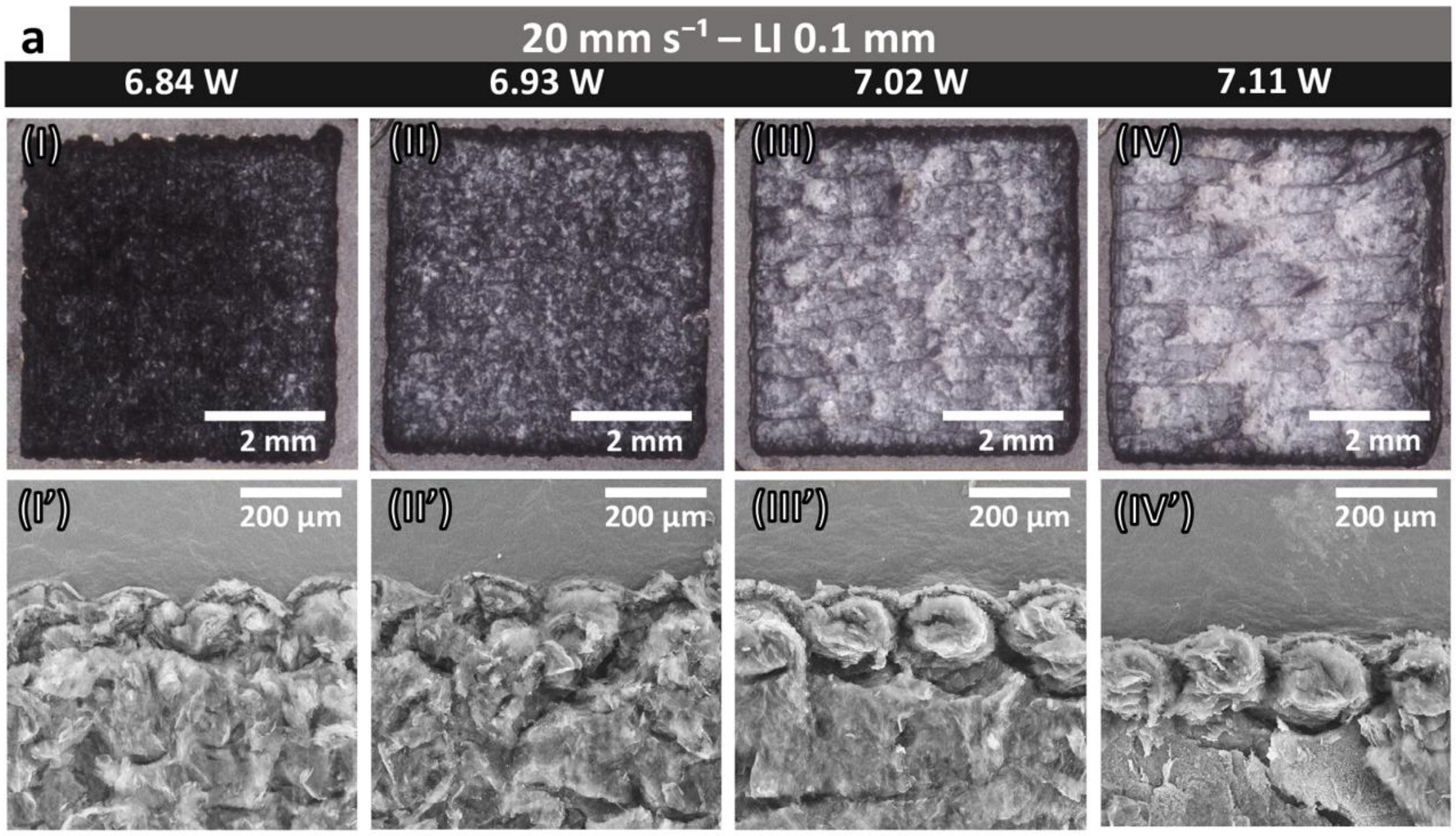

Figure S3. Morphological characterization of LrGO tracks as a function of Laser Power (LP), (6.84 – 7.11 W) at a fixed scan speed (SS) (20 mm s^{-1}) and line interval (LI) (0.1 mm). The panel displays digital photographs (I-IV), and corresponding top-view SEM micrographs (I'-IV'), demonstrating the influence of laser power on the surface reduction of the GO/CMC films.

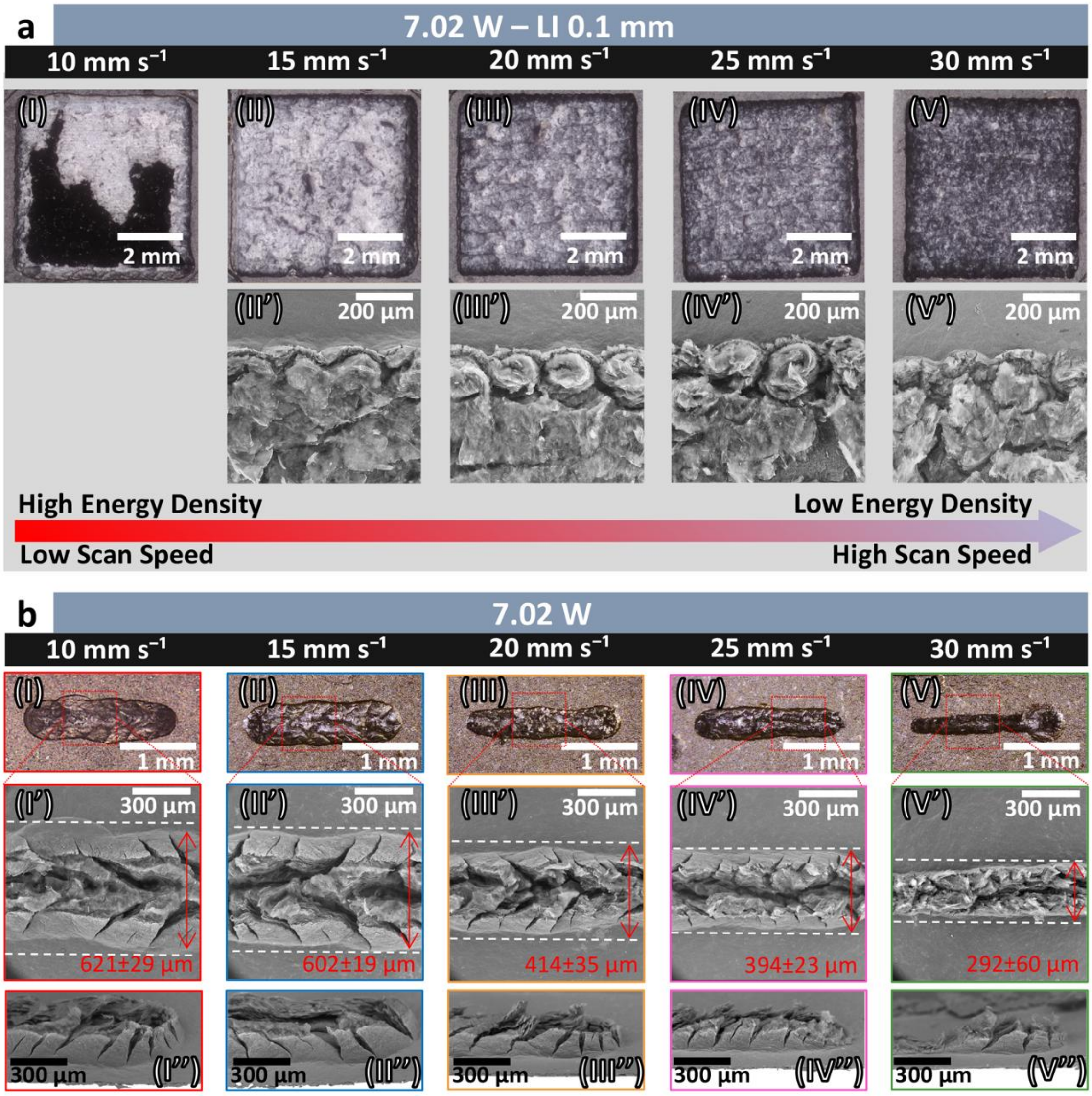

Figure S4. Comprehensive morphological characterization of LrGO tracks under different Scan speeds (SS), (10-30 mm s^{-1}) at a fixed LP (7.02 W) and LI (0.1 mm). **(a)** Digital photographs (I-V), and corresponding top-view SEM micrographs (I'-V'). **(b)** Detailed analysis of individual LrGO tracks, including digital photographs (I-V), top-view SEM (I'-V'), and cross-section SEM micrographs (I"-V") demonstrating the 3D structural expansion of the material from the GO/CMC matrix as a function of the delivered energy.

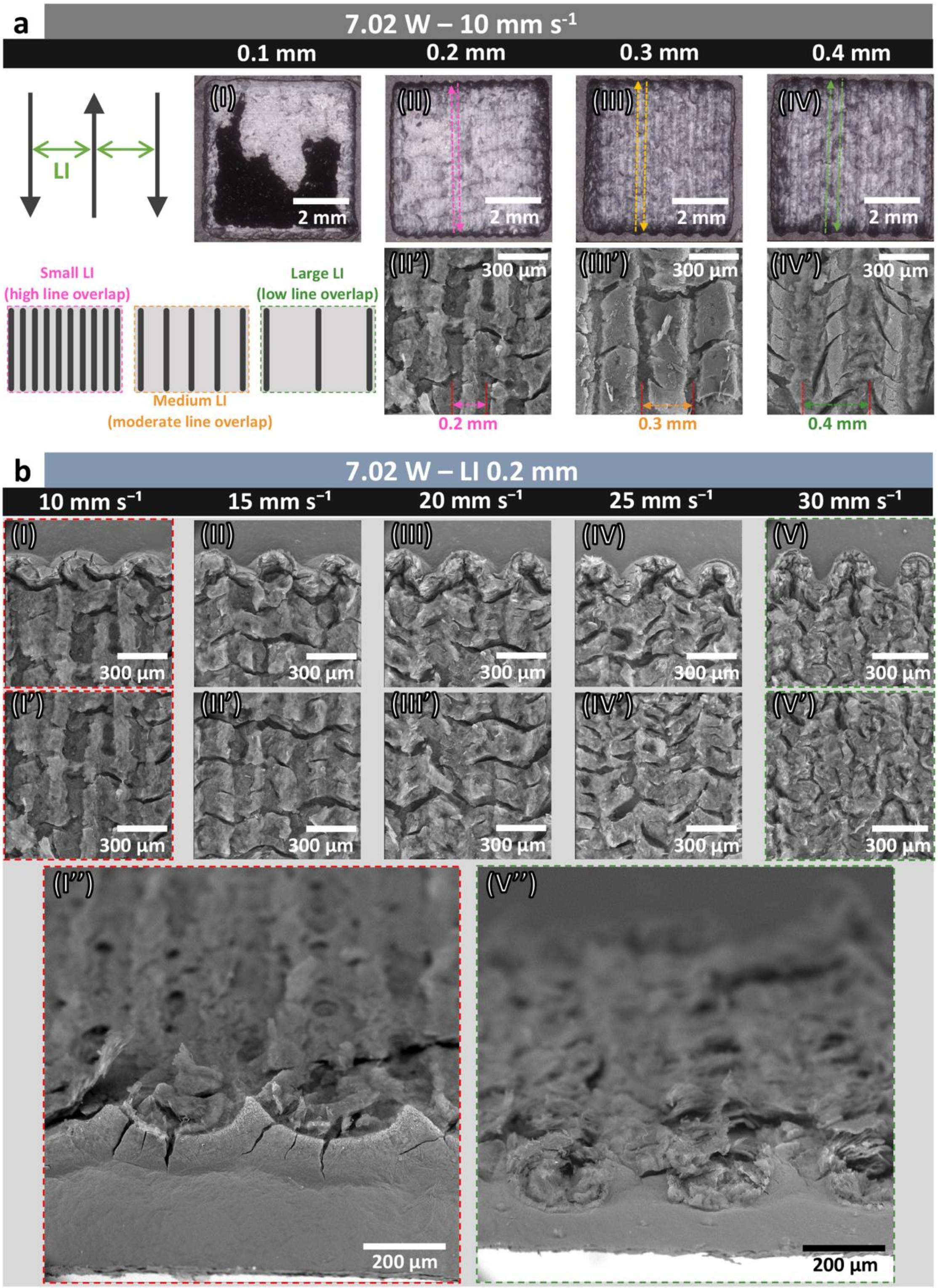

Figure S5. **(a)** Schematic representation of line interval (LI) and engraving direction effects on track overlap, complemented by the morphological characterization of LrGO tracks under different LI (0.1-0.4 mm) at fixed LP (7.02 W) and SS (10 mm s^{-1}). Digital photographs (I-IV) and corresponding top-view SEM micrographs (II'-IV'). **(b)** Morphological characterization of LrGO tracks as a function of scan speed (SS), (10-30

mm s^{-1}) at fixed LP (7.02 W) and LI (0.2 mm). The analysis presents digital photographs (I-V), top-view SEM micrographs (I'-V'), and cross-section SEM micrographs (I" for 10 mm s^{-1} and V" for 30 mm s^{-1}) demonstrating the impact of scan speed on 3D structural expansion and interconnectivity of the tracks.

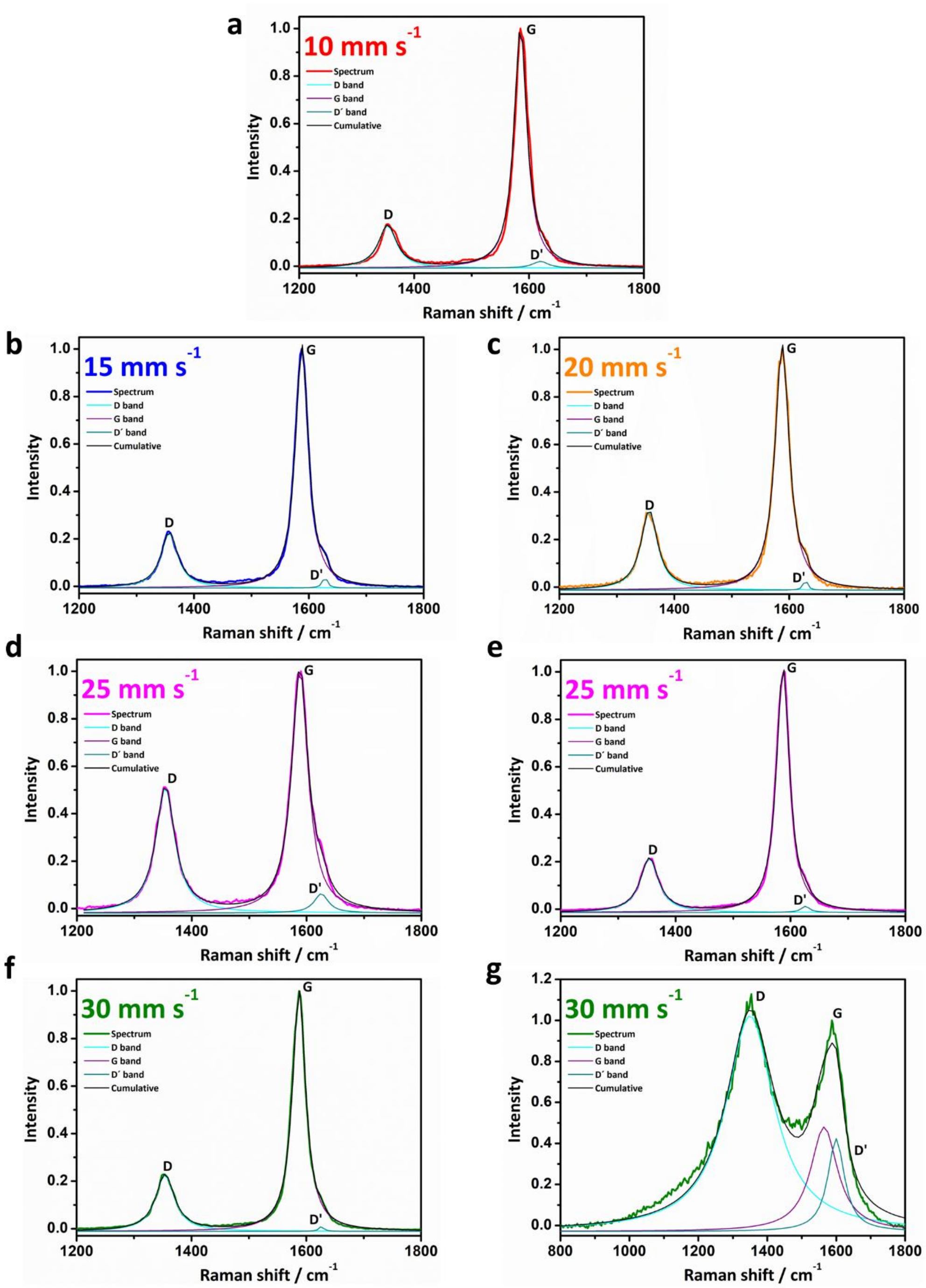

Figure S6. Deconvoluted Raman spectra of LrGO tracks fabricated at different scan speeds. **(a)** Spectrum at 10 mm s^{-1}, **(b)** 15 mm s^{-1}, **(c)** 20 mm s^{-1}, **(d)** profile A at 25 mm s^{-1}, **(e)** profile B at 25 mm s^{-1}, **(f)** profile A at 30 mm s^{-1}, and **(g)** profile B at 30 mm s^{-1}. The experimental data were fit to resolve the D and G bands for accurate I_D/I_G ratio calculation. For 25- and 30-mm s^{-1} conditions, two distinct spectral profiles (A and B) were identified and analyzed separately, reflecting the structural heterogeneity and incomplete reduction observed at higher processing speeds.

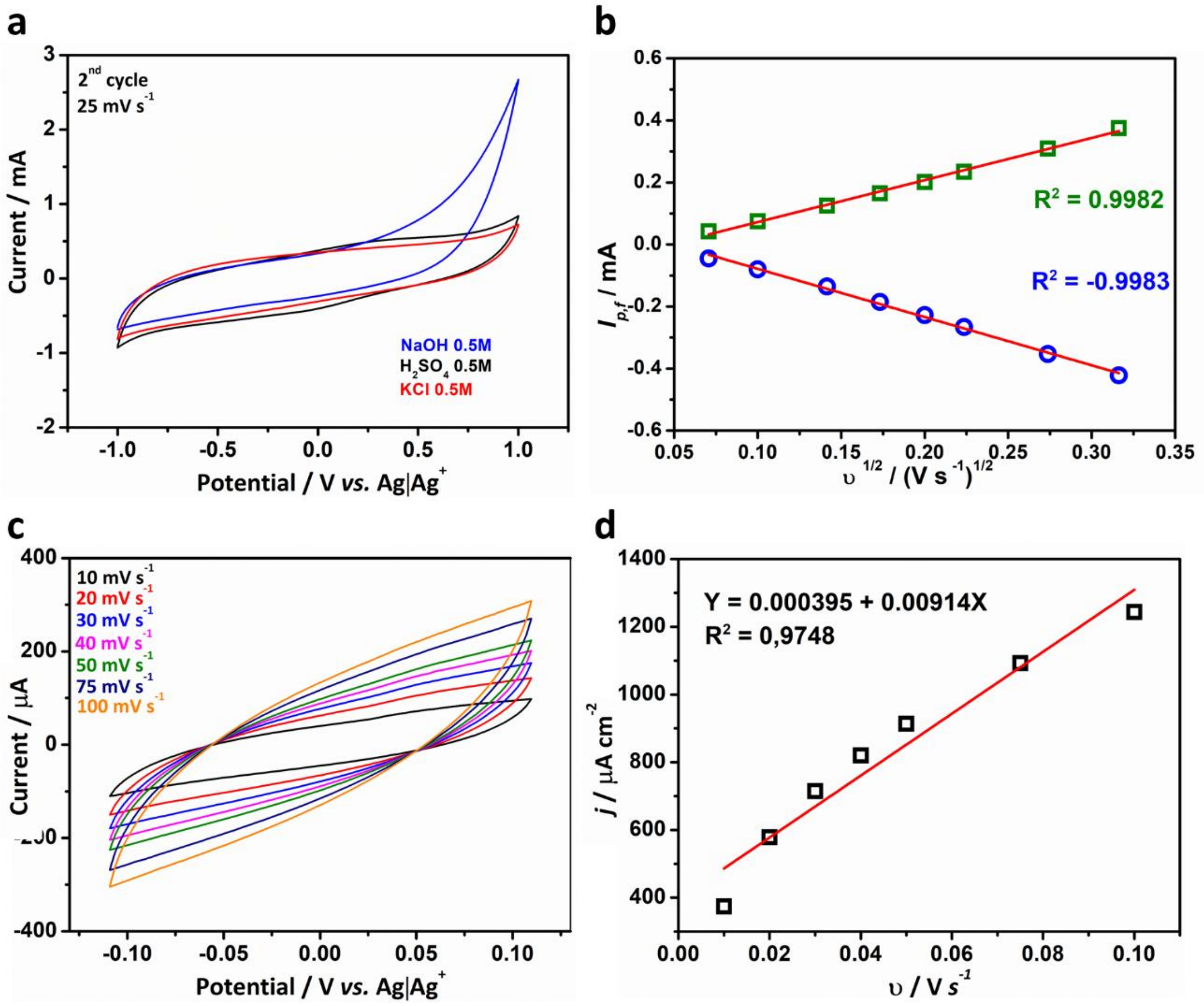

Figure S7. Electrochemical evaluation of capacitive behavior and electrochemically active surface area (ECSA). **(a)** Cyclic Voltammetry (CV) profiles recorded in different supporting electrolytes. **(b)** Linear correlation between anodic (I_{pa}) and cathodic (I_{pc}) peak currents and the square root of scan rate ($v^{1/2}$), derived from the $[Fe(CN)]^{3-/4-}$ redox probe analysis. **(c)** CV voltammograms recorded in the non-Faradaic region (0.5 M KCl) at scan rates ranging from 10 to 100 mV s^{-1}. **(d)** Linear plot of the anodic current density (j) versus scan rate (v), used to determine the double-layer capacitance (C_{dl}) and calculate the ECSA.

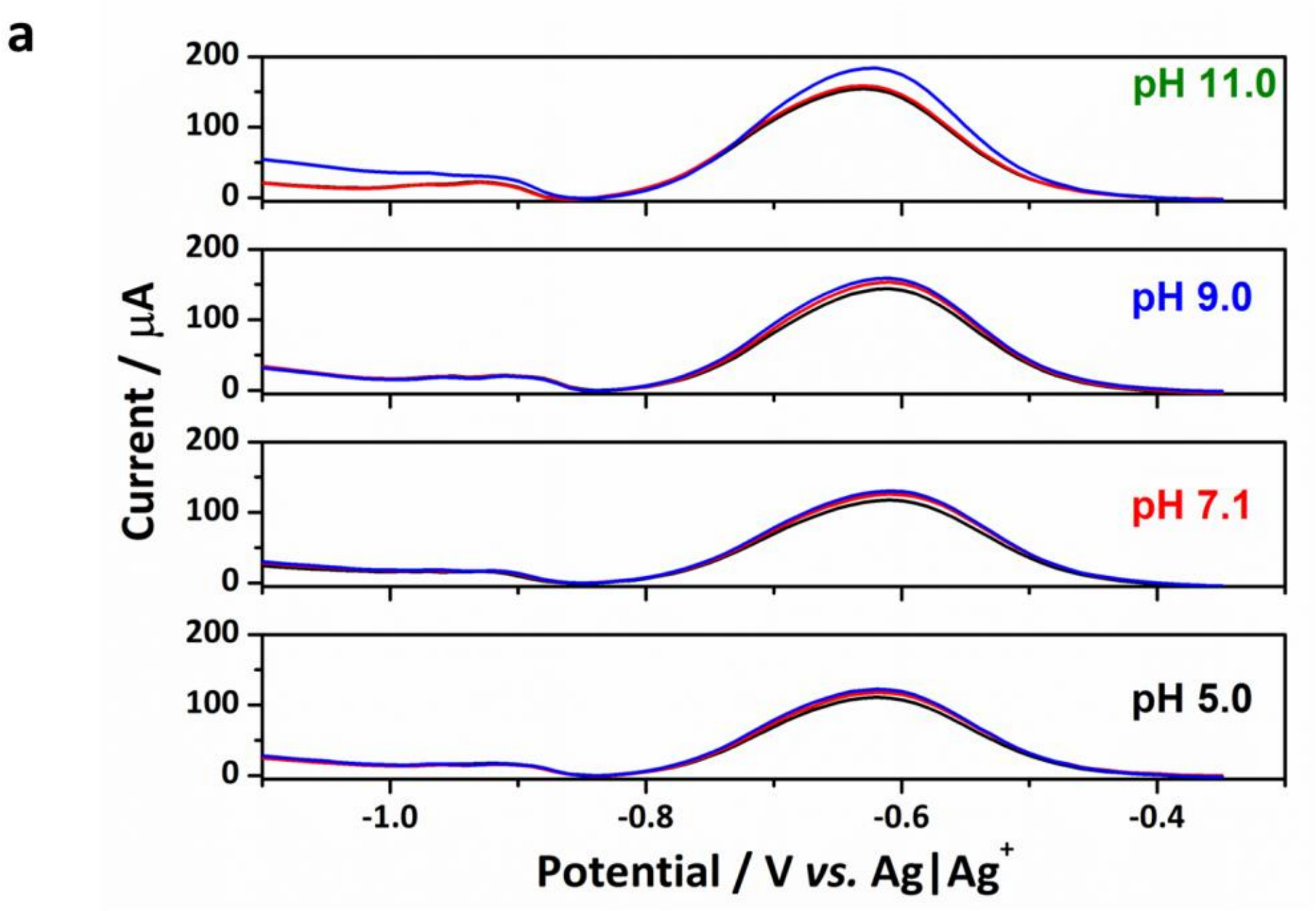

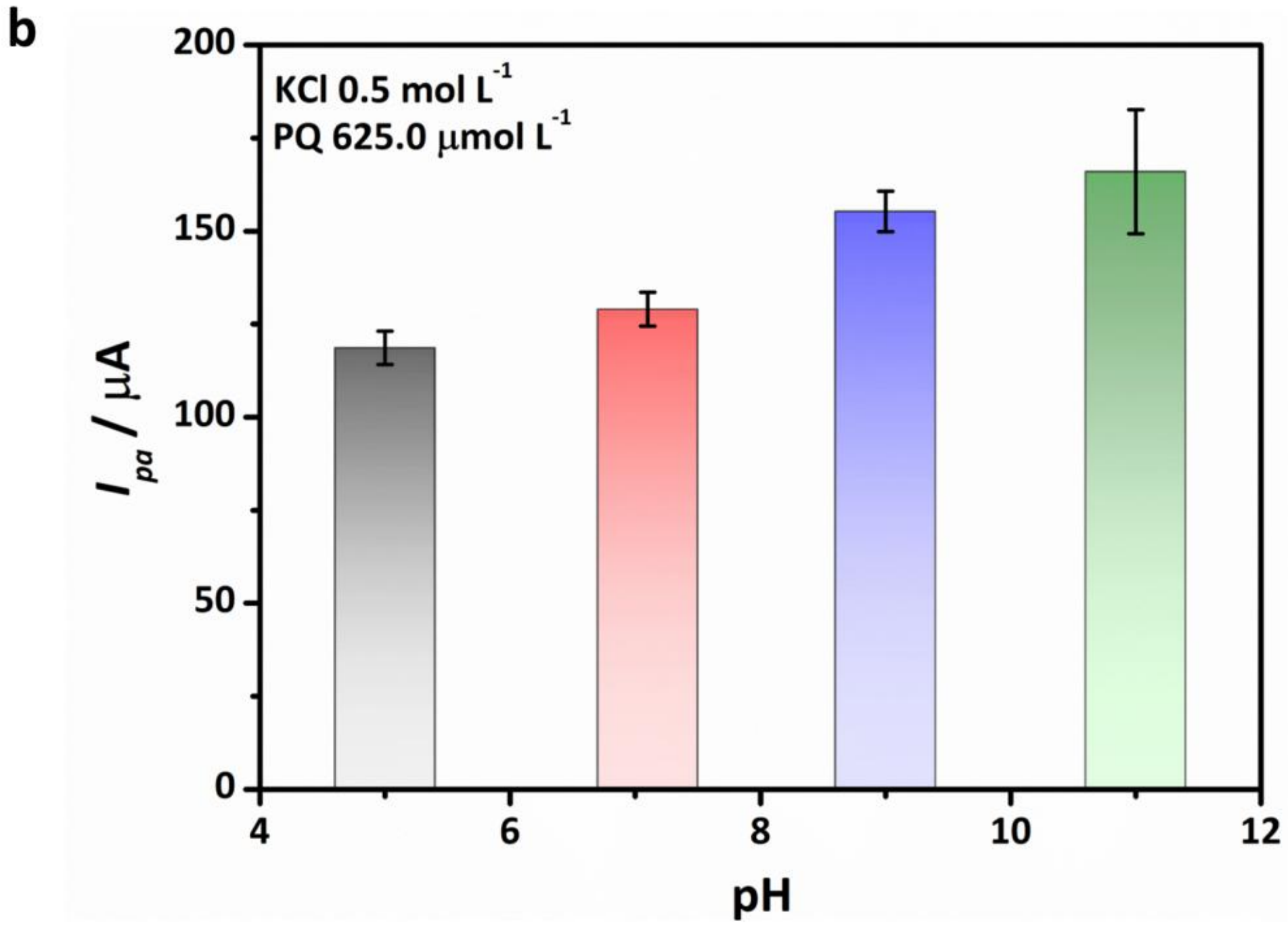

Figure S8. Evaluation of signal stability and reproducibility for Paraquat detection at different pH values. **(a)** Consecutive Differential Pulse Voltammetry (DPV) scans recorded at different pH values (5.0 – 11.0) in 0.5 mol L^{-1} KCl containing 625 µmol L^{-1} PQ. **(b)** Comparative plot of peak current intensity (I_{pa}) and corresponding Standard Deviation (SD) as a function of pH.

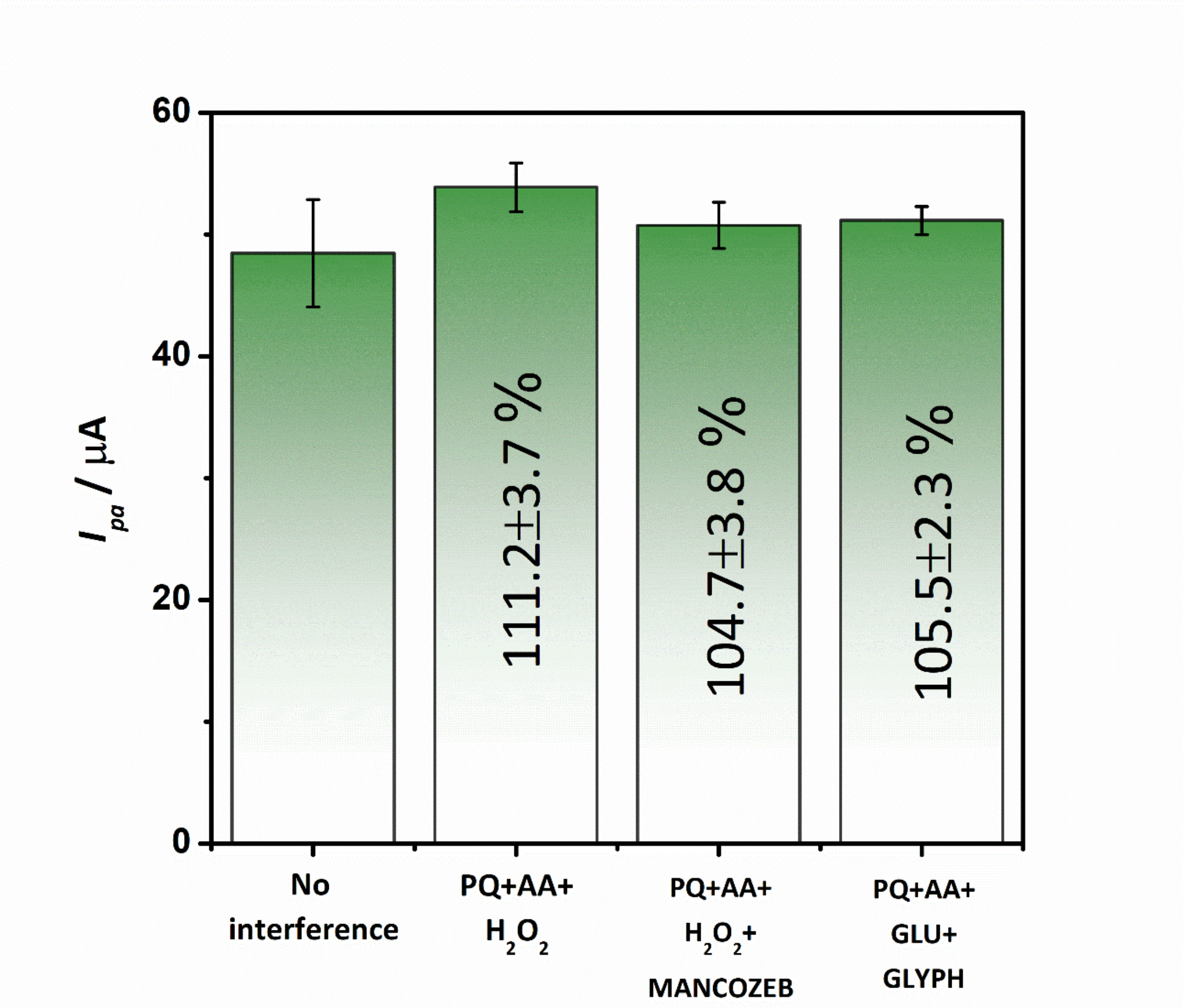

Figure S9. Selectivity profile of the LrGO/CMC sensor. The relative current of Paraquat (Control, 100%) is compared to the response obtained in the presence of complex interfering mixtures: (1) Ascorbic acid + H_2O_2; (2) Ascorbic acid + H_2O_2 + Mancozeb; and (3) Paraquat + Ascorbic acid + glucose + glyphosate. Error bars represent the standard deviation (n = 3).

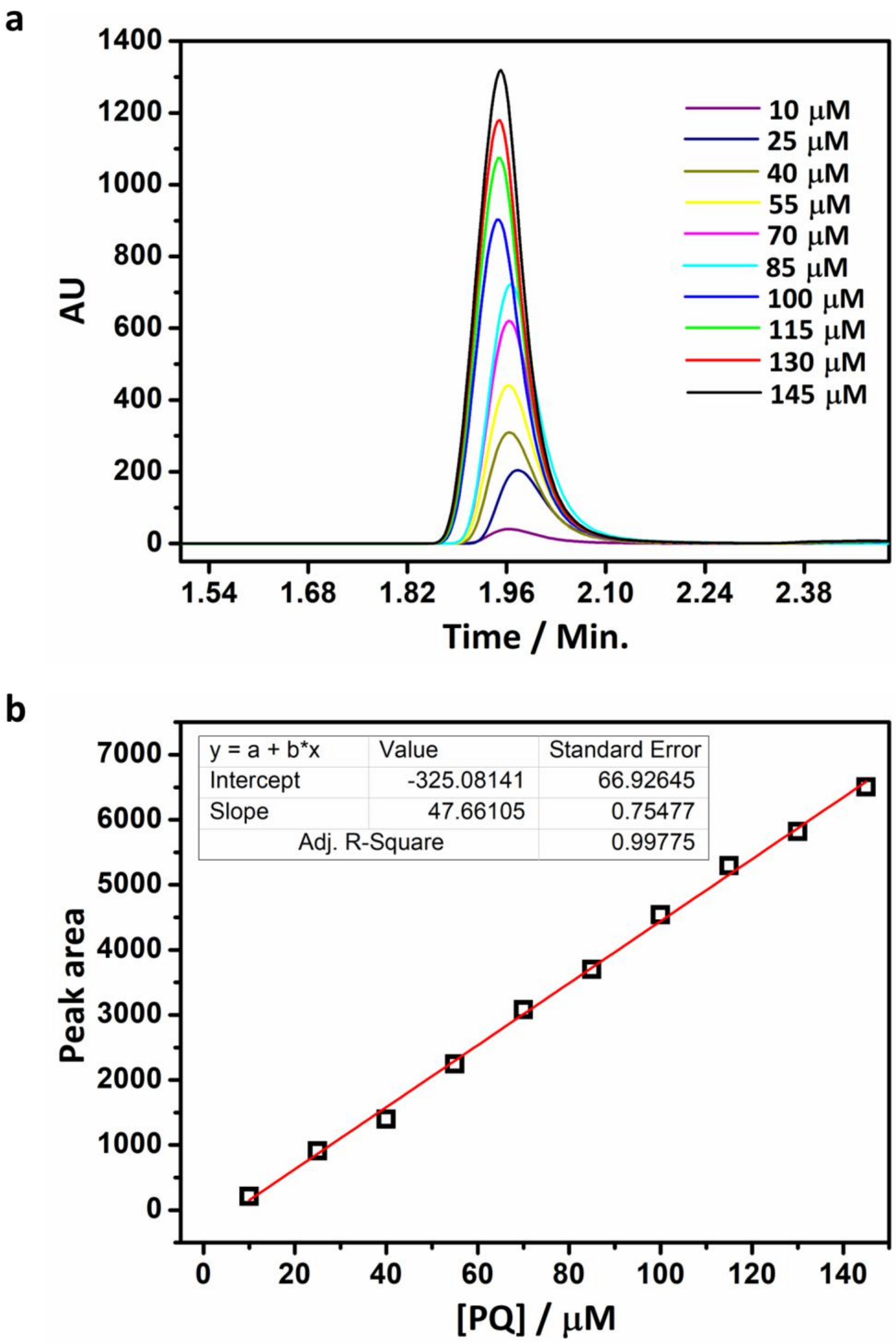

Figure S10. Analytical performance of the reference HPLC method for Paraquat (PQ) quantification. **(a)** HPLC chromatograms for increasing concentrations of PQ. **(b)** Corresponding calibration curve. The theoretical limits (LOD = 4.63 μmol L^{-1}; LOQ = 14.04 μmol L^{-1}) were established following the statistical criteria based on the intercept standard deviation and the slope.

Note S1. Calculation of In-Plane Crystallite Size (L_a)

The in-plane crystallite size (L_a) was estimated from the ratio of the integrated intensities of the D and G bands (I_D/I_G) obtained by Raman spectroscopy. According to the Tuinstra-Koenig relation, L_a is inversely proportional to the I_D/I_G ratio.[1, 2] The calculation was performed using Equation S1:

$$L_a\ (nm) = (2.4 \times 10^{-10})\lambda_l^4 \left(\frac{I_D}{I_G}\right)^{-1} \qquad \text{(Eq. S1)}$$

Where λ_l represents the wavelength of the excitation laser in nanometers, in this work, a laser with $\lambda_l = 532$ nm was used.

1. Cançado, L. G.; Takai, K.; Enoki, T.; Endo, M.; Kim, Y. A.; Mizusaki, H.; Jorio, A.; Coelho, L. N.; Magalhães-Paniago, R.; Pimenta, M. A., General equation for the determination of the crystallite size La of nanographite by Raman spectroscopy. *Applied Physics Letters* **2006,** *88* (16), 163106.
2. Tuinstra, F.; Koenig, J. L., Raman Spectrum of Graphite. *The Journal of Chemical Physics* **1970,** *53* (3), 1126-1130.
